# Tonsil volume and outcome of radiofrequency uvulopalatoplasty with or without tonsillectomy in adults with sleep-disordered breathing

**DOI:** 10.1007/s00405-023-07914-0

**Published:** 2023-03-12

**Authors:** Samuel Tschopp, Khalid Azalmad, Kurt Tschopp

**Affiliations:** 1 Department of Otorhinolaryngology, Head and Neck Surgery, Kantonsspital Baselland, Mühlemattstrasse 13, 4410 Liestal, Switzerland; 2 Department of Otorhinolaryngology, Head and Neck Surgery, Inselspital, University Hospital and University of Bern, Bern, Switzerland; 3 Faculty of Biology and Medicine, University of Lausanne, Lausanne, Switzerland

**Keywords:** Obstructive sleep apnea, Uvulopalatopharyngoplasty, Tonsillectomy, Palatine tonsil, Upper airway surgery

## Abstract

**Purpose:**

Predictors for the outcome of uvulopalatopharyngoplasty with and without tonsillectomy (UPPP ± TE) in sleep-disordered breathing have not been fully established. This study investigates tonsil grade, volume, and preoperative examination in predicting radiofrequency UPP ± TE outcomes.

**Methods:**

All patients undergoing radiofrequency UPP with tonsillectomy if tonsils were present between 2015 and 2021 were retrospectively analyzed. Patients underwent a standardized clinical examination, including Brodsky palatine tonsil grade from 0 to 4. Preoperatively and 3 months after surgery, sleep apnea testing was performed using respiratory polygraphy. Questionnaires were administered assessing daytime sleepiness using the Epworth Sleepiness Scale (ESS) and snoring intensity on a visual analog scale. Tonsil volume was measured intraoperatively using water displacement.

**Results:**

The baseline characteristics of 307 patients and the follow-up data of 228 patients were analyzed. Tonsil volume increased by 2.5 ml (95% CI 2.1–2.9 ml; *P* < 0.001) per tonsil grade. Higher tonsil volumes were measured in men, younger patients, and patients with higher body mass indices. The preoperative apnea–hypopnea index (AHI) and AHI reduction strongly correlated with tonsil volume and grade, whereas postoperative AHI did not. The responder rate increased from 14% to 83% from tonsil grade 0 to 4 (*P* < 0.01). ESS and snoring were significantly reduced after surgery (*P* < 0.01), but the reduction was not influenced by tonsil grade or volume. No other preoperative factor other than tonsil size could predict surgical outcomes.

**Conclusions:**

Tonsil grade and intraoperatively measured volume correlate well and predict the reduction of AHI, while they are not predictive of ESS and snoring response after radiofrequency UPP ± TE.

**Supplementary Information:**

The online version contains supplementary material available at 10.1007/s00405-023-07914-0.

## Introduction

Obstructive sleep apnea (OSA) affects a large proportion of the adult population. One study estimates that 23% of women and 49% of men suffer from OSA, defined as 15 or more apneas and hypopneas per hour (AHI) [1]. Several studies could demonstrate long-term complications of untreated OSA, such as cardiovascular disease, stroke, and increased mortality [2, 3]. Continuous positive airway pressure therapy is the main treatment option, but 19–49% of patients do not adhere to this treatment [4].

Several surgical techniques have been developed as alternative treatments. The most frequently performed surgery is uvulopalatopharyngoplasty with or without tonsillectomy (UPPP ± TE) [5]. In 1981, Fujita et al. first described the procedure, which was subsequently modified and refined [6]. In a randomized controlled trial, UPPP with TE proved superior in reducing AHI compared to no intervention [5]. Stuck et al. performed a meta-analysis and found a good treatment effect of UPPP ± TE on respiratory events and daytime sleepiness [7].

Palatine tonsil size is a well-established risk factor for OSA, and multiple studies have correlated tonsil grade with tonsil volume measured in tonsillectomy specimens [8–10]. However, the surgical outcome of UPPP ± TE depending on tonsil size and volume has not been comprehensively investigated, despite indications that it is an important predictor of successful surgery. In a previous pilot study, we demonstrated that tonsil size and volume are good predictors of postoperative reduction of respiratory events but not for snoring or daytime symptoms [11]. Matarredona et al. could partially replicate our findings for tonsil volume but failed to find an association between tonsil grade and successful outcomes [12]. Both studies included only a small cohort leaving many outcome parameters uninvestigated or statistically underpowered.

In this study, we extend our previous analysis on the impact of tonsil size and volume on the outcome of radiofrequency uvulopalatoplasty ± TE (rfUPP ± TE) on respiratory parameters and symptoms. The larger cohort with more detailed clinical examination parameters allows the investigation of other factors predicting the surgical outcome, which many authors have proposed [9, 13]. To our knowledge, this is the most extensive study investigating the effect of tonsil size, volume, and clinical predictors on the outcome of rfUPP ± TE.

## Materials and methods

A retrospective analysis of all rfUPP ± TE surgeries at our institution between 2015 and 2021 was performed. Patients were offered rfUPP ± TE based on history, physical examination, and respiratory polygraphy. All severities of sleep-disordered breathing, including habitual snoring, were analyzed. We excluded patients with significant missing data, concomitant surgery other than nasal surgery, or aged less than 18 years. This study was conducted according to the Declaration of Helsinki and approved by the Swiss ethics committee (EKNZ 2021-02324). The reporting follows the Strengthening the Reporting of Observational Studies in Epidemiology (STROBE) guideline [14].

### Data collection

Before surgery, all patients underwent an extensive examination of the upper airway, summarized on a standardized report form. Tonsil size was graded using the system proposed by Brodsky and the tongue position according to Friedman [15, 16]. In the case of tonsil asymmetry, the larger side was used for clinical grading. Home sleep apnea testing and symptoms questionnaires were performed preoperatively and 3 months postoperatively. Daytime sleepiness was reported using the Epworth Sleepiness Scale (ESS), depression symptoms using the Beck Depression Inventory (BDI), insomnia symptoms using the Insomnia Severity Index (ISI), and snoring intensity (SI) on a visual analog scale from 0 to 10 [17–19]. We recorded adverse events and patients’ satisfaction with the questions of whether they would undergo surgery again or recommend it to a friend.

### Surgery

Radiofrequency UPP ± TE was performed in general anesthesia with radiofrequency ablation of the soft palate and incision of the palatopharyngeal arch to release tension. Radiofrequency ablation was administered by inserting a bipolar electrode into the soft palate four to five times per side until a visible stiffening and contraction were achieved and deemed sufficient by the surgeon. Overly long uvulae (> 12 mm) were shortened. All patients with tonsils underwent a cold-steel tonsillectomy, irrespective of tonsil grade and OSA severity. Removing tonsils, if present, was performed to stabilize the lateral pharyngeal wall and increase upper airway volume. The volume of tonsillectomy specimens was measured using Archimedes’ principle of water displacement, and the sum of both tonsil volumes was used for statistical analysis.

### Statistical analysis

Statistical analyses were performed in RStudio using R (R Project for Statistical Computing, Version 4.2.0) with the consultation of the Clinical Trial Unit Basel. Tonsil volume was compared between tonsil grades using linear and multiple regression to adjust for confounding factors. The primary endpoint for surgical outcomes was the AHI’s absolute and relative reduction. Secondary endpoints were the effects of rfUPP ± TE on oxygen desaturation index (ODI), ESS, and SI. The Sher criteria, with a postoperative AHI ≤ 20/h and ≥ 50% reduction from baseline, were used to define success for respiratory parameters [20]. Likewise, the resolution of daytime sleepiness was regarded as an ESS value ≤ 10 and a reduction of ≥ 50% from preoperative. Successful treatment of snoring was defined as a value ≤ 3 and a reduction ≥ 50% on a visual analog scale. Categorical variables were compared using Fisher’s exact or Chi-squared test with continuity correction. After testing for normality, continuous variables were compared using *t* test and ANOVA for normal or Wilcoxon and Kruskal–Wallis test for non-normal distributed variables. Multiple linear and logistic regressions were used to identify co-factors for score reduction and success. For patients lost to follow-up, baseline characteristics were compared to those included to investigate a systematic bias due to attrition. Statistical tests were performed two-sided, and *P* values < 0.05 were considered statistically significant.

## Results

In total, 354 patients underwent palatal surgery for sleep-disordered breathing at our institution. Six patients were excluded for missing head and neck examination and 41 for missing tonsil volume documentation, leaving 307 patients included in this study and analyzed for the baseline characteristics. For the follow-up, we excluded one patient for concomitant hyoid suspension and 78 patients for missing follow-up data, leaving 228 patients to be analyzed postoperatively. The median follow-up was 100 days (interquartile range 90–135 days). Patient characteristics are summarized by tonsil grade in Table 1.

**Table 1:** Baseline characteristics of all patients and by tonsil grade given as mean and standard deviations in round brackets or median and interquartile range in square brackets

	Overall	Tonsil grade					*P* value
		0	1	2	3	4	
No. of patients	307	12	75	150	62	8	
Tonsil volume (mL)	6.1 (3.6)	0 (0)	4.5 (2.2)	5.9 (2.5)	8.9 (4.0)	12.6 (5.7)	< 0.01
Gender, female	36 (11.7)	1 (8.3)	11 (14.7)	16 (10.7)	6 (9.7)	2 (25.0)	0.56
Age, years	44.6 (11.5)	46.4 (10.7)	47.2 (10.6)	45.2 (10.5)	40.3 (12.9)	39.5 (16.8)	< 0.01
Height (cm)	177.1 (8.4)	180.7 (11.4)	177.2 (9.7)	176.8 (7.2)	177.6 (8.8)	173.0 (8.1)	0.36
Weight (kg)	89.9 (15.6)	93.1 (11.3)	87.0 (12.9)	89.5 (15.1)	92.1 (15.4)	97.2 (37.9)	0.23
Body mass index (kg/m^2^)	28.6 (4.5)	28.6 (3.3)	27.7 (3.2)	28.6 (4.4)	29.3 (4.8)	32.0 (10.7)	0.07
Neck circumference (cm)	41.6 (3.8)	45.0 (NA)	40.7 (3.7)	41.7 (3.7)	42.1 (3.9)	45.0 (2.8)	0.15
Preoperative							
Apnea–hypopnea index	23.9 (18.8)	16.7 (8.9)	21.9 (16.4)	22.4 (18.1)	30.6 (22.3)	31.1 (22.1)	0.01
Epworth Sleepiness Scale	8.6 (4.8)	5.5 (4.8)	8.1 (5.0)	8.7 (4.7)	9.3 (4.3)	9.8 (6.5)	0.12
Snoring index (VAS 0–10)	8.0 [7.0, 9.0]	7.0 [7.0, 8.5]	8.0 [8.0, 9.0]	8.0 [7.0, 9.8]	9.0 [7.0, 9.5]	8.5 [8.0, 9.2]	0.51
No. of follow-up patients	228	7	60	113	42	6	
AHI postoperative	16.1 (14.9)	19.1 (13.5)	16.9 (14.0)	15.6 (14.6)	16.8 (17.7)	6.8 (4.6)	0.55
AHI reduction	10.0 (16.9)	-0.4 (10.1)	6.6 (14.7)	9.4 (16.2)	16.5 (18.9)	25.6 (22.6)	< 0.01
AHI Responder	96 (41.9)	1 (12.5)	18 (30.0)	48 (42.5)	24 (57.1)	5 (83.3)	0.01
ESS postoperative	3.9 (2.9)	NA	3.9 (3.2)	4.3 (3.0)	3.0 (1.9)	2.7 (1.2)	0.19
ESS reduction	4.8 (4.7)	NA	4.0 (4.9)	4.6 (4.6)	6.0 (4.0)	11.3 (4.0)	0.03
ESS responder	78 (59.5)	0 (NA)	21 (55.3)	36 (54.5)	18 (75.0)	3 (100.0)	0.16
SI postoperative	3.0 [2.0, 4.0]	NA	3.0 [2.0, 5.0]	3.0 [2.0, 4.0]	3.0 [2.0, 4.0]	3.0 [2.5, 3.5]	0.88
SI reduction	5.0 [4.0, 6.2]	NA	5.0 [3.8, 6.2]	5.0 [3.0, 6.0]	6.0 [4.2, 7.0]	6.0 [6.0, 6.0]	0.31
SI responder	61 (54.5)	0 (NA)	19 (55.9)	25 (47.2)	16 (69.6)	1 (50.0)	0.3

Significance testing between tonsil grades using chi-square, Fisher’s exact, ANOVA or Mann–Whitney *U* test

*AHI* apnea–hypopnea index, *ESS* Epworth Sleepiness Scale, *SI* snoring index (VAS 0–10)

### Tonsil volume and grade

**Fig. 1:**
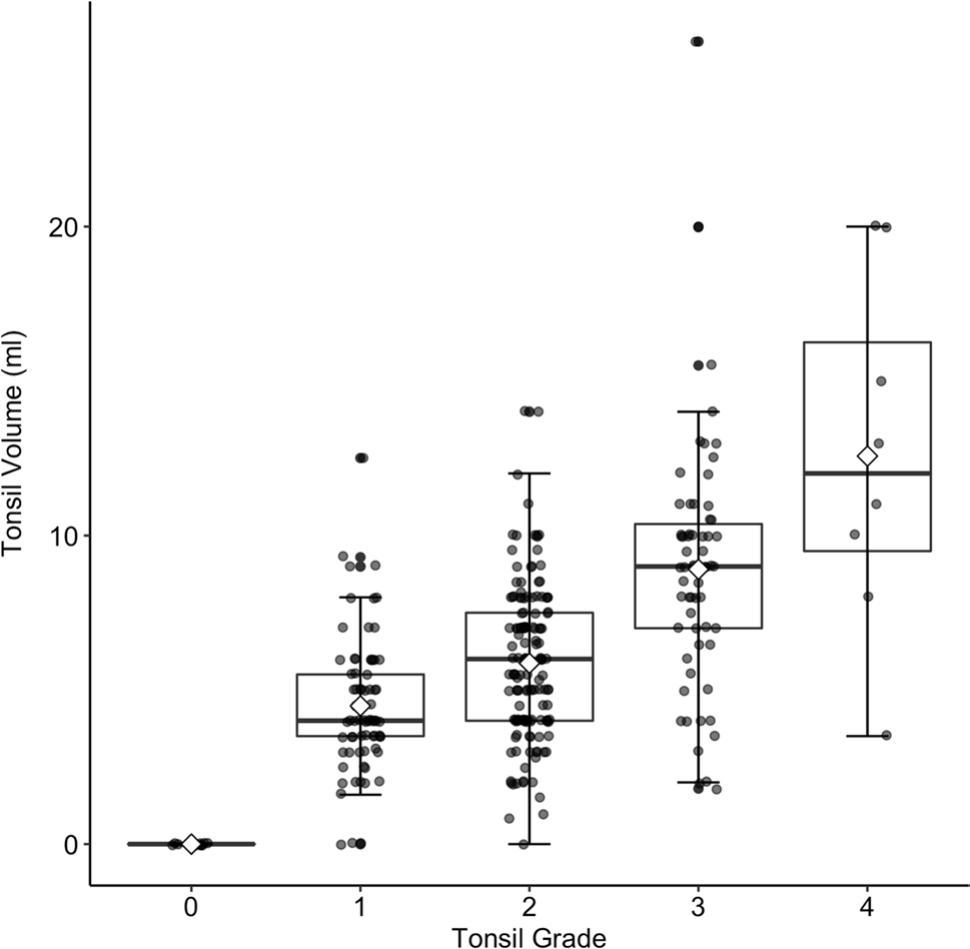
Palatine tonsil volume increases with tonsil grade according to Brodsky. Each tonsil grade is given as a boxplot with the mean indicated by a diamond shape. After adjusting for sex, age, and BMI, tonsil volume increased by 2.4 ml (95% CI 1.9–2.8 ml; *P* < 0.001) per tonsil grade (*n* = 307)

Tonsil volume significantly increased with each tonsil grade (Fig. 1, Table 1). Tonsil volume was significantly higher in men than in women (6.2 ± 3.7 ml vs. 4.9 ± 2.9 ml, *P* = 0.02), decreased with age by 0.09 ml per year (95% CI 0.05–0.12 ml per year; *P* < 0.001), and increased with BMI by 0.10 ml per 1 kg/m^2^ (95% CI 0.001–0.20 ml; *P* = 0.05). No other patient characteristic significantly influenced tonsil volume. A linear regression model showed a tonsil volume increase of 2.5 ml (95% CI 2.14–2.92 ml; *P* < 0.001) for each grade. After adjusting for sex, age, and BMI, tonsil volume increased by 2.4 ml (95% CI 1.9–2.8 ml; *P* < 0.001) per tonsil grade.

### Apnea–hypopnea index (AHI)

**Fig. 2:**
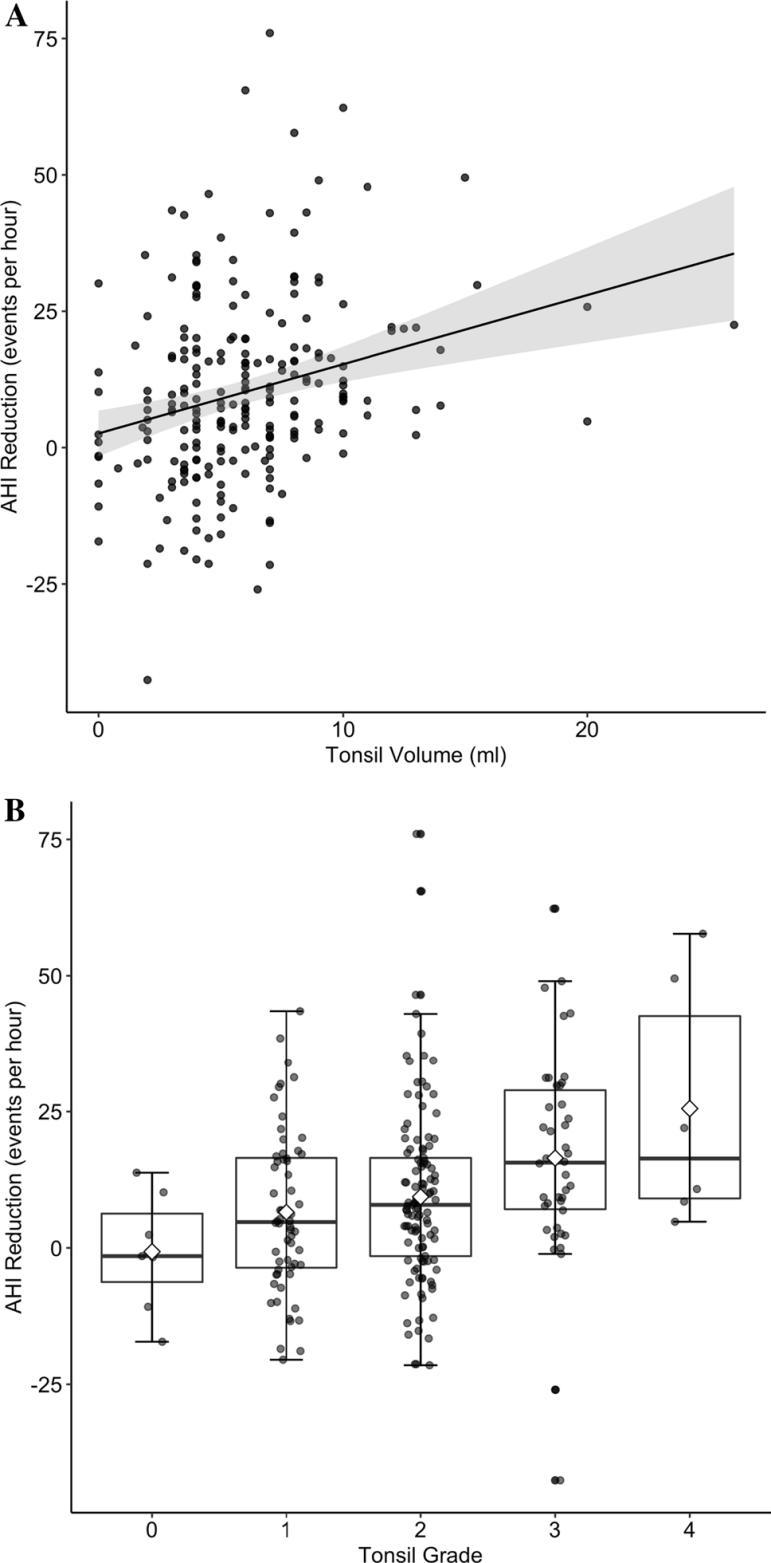
Apnea–hypopnea reduction increases with palatine tonsil grade (**A**) and tonsil volume (**B**). Figure A displays a linear regression with a 95% confidence interval. A diamond shape indicates the mean in Figure B. The absolute AHI reduction increased with volume by 1.3/h per mL (95% CI 0.7/h–1.9/h; *P* < 0.001) and tonsil grade by 5.4/h per grade (95% CI 2.8/h–8.0/h per grade; *P* < 0.001) (*n* = 228)

**Fig. 3:**
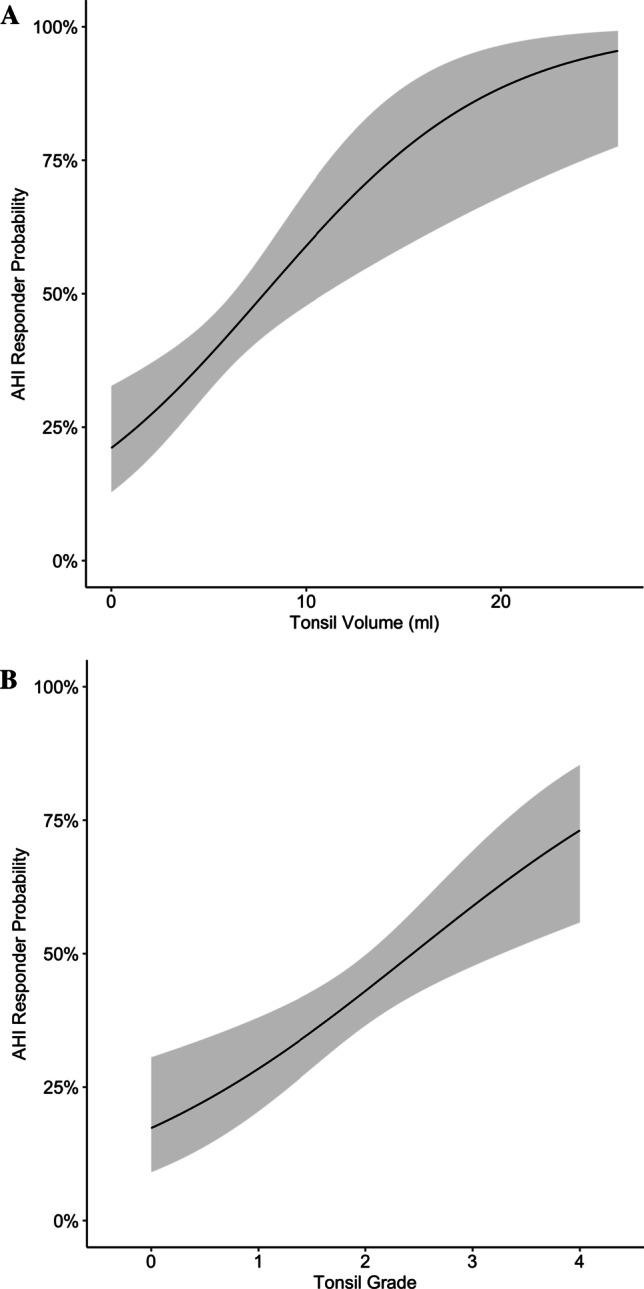
Logistic model for responder according to Sher by tonsil volume (**A**) and tonsil grade (**B**) with 95% confidence interval, indicating an increased odds ratio for success with larger tonsils (*n* = 228)

Preoperative AHI increased with tonsil grade and tonsil volume. Linear regression showed preoperative AHI to increase by 4.0/h (95% CI 1.5/h–6.4/h; *P* = 0.002) for every grade and by 0.8 (95% CI 0.2/h–1.3/h; *P* = 0.01) per ml of total tonsil volume (see Online Resource 1 and 2). Postoperative AHI values were not significantly influenced by tonsil volume or grade (*P* = 0.07 and 0.55, respectively). The absolute AHI reduction increased with volume by 1.3/h per mL (95% CI 0.7/h–1.9/h; *P* < 0.001) and tonsil grade by 5.4/h per grade (95% CI 2.8/h–8.0/h per grade; *P* < 0.001), see Fig. 2 and Table 2. This relationship was also significant for the relative AHI reduction increasing by 21.8 ± 7.9% per grade (*P* = 0.006) and 5.9 ± 1.8% per ml (*P* = 0.006). After controlling for age, BMI, and neck circumference, tonsil volume, and grade were still significantly associated with preoperative AHI and the reduction through surgery. The tonsil volume of responders, according to Sher, was significantly higher than that of non-responders (7.0 ± 4.1 mL vs. 5.2 ± 2.8 mL, *P* < 0.001, see Online Resource 3). From tonsil grade 0 to 4, the rate of AHI responders increased from 14% (1/7), 30% (18/60), 42% (48/113), 57% (24/42) to 83% (5/6), respectively (*P* = 0.008). The increased rate for responders by tonsil volume and grade are shown in the logistic models in Fig. 3. A comparison of response by preoperative OSA severity is given in Table 3.

**Table 2:** Comparison of pre- and postoperative sleep studies (*n* = 228)

	Preoperative	Postoperative	Absolute difference	Relative difference (%)	*P* value
Recording time (hours)	7.2 (1.4)	7.4 (1.1)	0.2	2.7	0.12
Sleep time (hours)	6.0 (1.5)	6.1 (1.3)	0.1	1.3	0.45
AHI	26.1 (18.9)	16.1 (14.9)	− 10.1	− 38.6	< 0.01
ODI	20.9 (18.9)	13.3 (12.4)	− 7.6	− 36.4	< 0.01
AI	10.9 (14.7)	4.4 (7.8)	− 6.5	− 59.7	< 0.01
Central AHI	2.3 (4.0)	0.9 (1.6)	− 1.4	− 61	0.05
AHI supine	37.1 (24.6)	26.3 (23.8)	− 10.8	− 29.1	< 0.01
ODI supine	31.33 (24.5)	22.1 (19.8)	− 9.1	− 29.2	< 0.01
AI supine	20.8 (24.2)	10.2 (17.5)	− 10.6	− 51.1	< 0.01
Cartwright Index	1.8 (1.6)	1.9 (1.9)	0.1	5	0.78
Supine time (% of total sleep time)	38.7 (26.4)	39.5 (25.7)	0.7	1.9	0.07
Mean oxygen saturation (%)	93.1 (2.0)	93.2 (1.9)	0.1	0.1	0.78
Mean oxygen desaturation (%)	90.1 (3.8)	90 (4.3)	− 0.1	− 0.1	0.37
Time below 90% oxygen saturation (minutes)	7.3 (13.8)	5.7 (11.8)	− 1.6	− 21.4	0.16
Heart rate (beats/minute)	63.2 (7.3)	64.3 (8.1)	1.1	1.7	0.01
Snoring Index (VAS 0–10)	8 [7, 9]	3 [2, 4]	− 5	− 62.5	< 0.01
Epworth Sleepiness Scale	9.1 (4.8)	3.9 (2.9)	− 5.2	− 57.4	< 0.01
Insomnia Severity Scale	13.6 (6.2)	7.7 (5.6)	− 5.8	− 42.4	0.03
Beck Depression Inventory	6 [2, 11]	6.5 [0, 8]	0.5	− 8.3	0.83

*AHI* apnea–hypopnea index, *ODI* oxygen desaturation index, *AI* apnea index

**Table 3:** Response to surgery stratified by preoperative sleep apnea severity defined as no OSA (AHI < 5), mild OSA (AHI 5–15), moderate OSA (AHI 15–30), and severe OSA (AHI > 30) (*n* = 228)

OSA severity	AHI preoperative	AHI postoperative	AHI change	AHI responder	ESS responder	Snoring responder
no	3.2 ± 1.4	8.3 ± 4.9	5.1 ± 5.1	not applicable	16.7% (1/6)	50% (3/6)
mild	10.7 ± 2.9	10.2 ± 7.8	− 0.4 ± 7.9	25.8% (17/66)	57.1% (16/28)	52% (13/25)
moderate	21.5 ± 4.5	13.3 ± 10.8	− 8.2 ± 10.9	53.2% (41/77)	65.9% (29/44)	51.5% (17/33)
severe	48.5 ± 16.1	25.3 ± 19.5	− 23.2 ± 20.3	52.1% (38/73)	70% (28/40)	65.7% (23/35)

*AHI* apnea–hypopnea index, *ESS* Epworth Sleepiness Scale

### Oxygen Desaturation Index (ODI)

Preoperative ODI increased by 4.0/h (95% CI 1.1/h–6.8/h; *P* = 0.006) per grade and by 0.8/h (95% CI 0.2/h–1.4/h; *P* = 0.01) per ml of total tonsil volume. Postoperative ODI was not significantly influenced by tonsil grade (*P* = 0.87) or tonsil volume (*P* = 0.43). The absolute ODI reduction increased for every tonsil grade by 5.1/h (95% CI 2.3/h–8.0/h; *P* < 0.001) and by 0.9/h (95% CI 0.3/h–1.5/h; *P* = 0.006) per ml of total tonsil volume. Similarly, the relative ODI reduction increased by 41.0 ± 18.6% (*P* = 0.03) and 5.6 ± 4.0% (*P* = 0.006) per grade and ml total volume, respectively.

### Epworth Sleepiness Scale (ESS)

Excessive daytime sleepiness (ESS > 10) was reported by 35% (99/283) of patients before and 1.5% (2/135) after surgery. 60% fulfilled the ESS responder criteria. Patients who responded to treatment regarding ESS had significantly larger tonsils compared to non-responders, 6.5 ± 3.4 ml vs. 6.2 ± 3.8 ml (*P* < 0.001), albeit the absolute volume difference was small. Preoperative and postoperative ESS was not significantly different among tonsil grades (*P* = 0.12 and 0.19, respectively, Table 1). There was also no significant association of tonsil volume with pre- or postoperative ESS (*P* = 0.06 and 0.43, respectively). The absolute reduction in the ESS was neither correlated with tonsil grade (*P* = 0.06) nor tonsil volume (*P* = 0.44).

### Snoring

Reported snoring intensity decreased from 8 [7–9] to 3 [2–4] (*P* < 0.001), with 54% of patients (61/112) fulfilling the responder criteria. No association was found between tonsil grade or volume and preoperative, postoperative, or reduction in snoring. However, responders for snoring had significantly larger tonsils with 6.5 ± 3.3 ml compared to 6.0 ± 2.7 ml for non-responders (*P* < 0.001).

### Co-factors

We further explored the data for predictors of outcome.

Sex did not significantly affect the pre- and postoperative value nor changes in AHI, ODI, ESS, and snoring.

Age was positively correlated with pre- and postoperative AHI with an increase of 3.8 ± 0.1/h (adjusted *R*^2^ = 0.04, *P* < 0.001) and 3.6 ± 0.1/h (adjusted *R*^2^ = 0.07, *P* < 0.001) for every year, respectively. The reduction through surgery was not influenced by age (*P* = 0.54).

Neck circumference correlated positively with pre- (1.3 ± 0.4/h per cm, adjusted *R*^2^ = 0.06, *P* < 0.001) and postoperative (1.2 ± 0.3/h per cm, adjusted *R*^2^ = 0.07; *P* < 0.001) AHI, but not the AHI reduction (*P* = 0.63).

BMI positively correlated with pre- (adjusted *R*^2^ = 0.06; *P* < 0.001) and postoperative (adjusted *R*^2^ = 0.06; *P* < 0.001) AHI. The reduction through surgery did not depend on BMI (*P* = 0.54).

Positional OSA, as measured by the Cartwright index, was inversely correlated with preoperative AHI (− 4.2 ± 1.0, adjusted *R*^2^ = 0.08;* P* < 0.001) and AHI reduction (− 2.8 ± 0.9, adjusted *R*^2^ = 0.04; *P* < 0.003), without a significant correlation with postoperative AHI (*P* = 0.08).

Insomnia symptoms slightly improved after surgery without reaching significance. On the Insomnia Severity Index, patients reported 12.5 ± 6.4 and 7.7 ± 5.6, pre- and postoperatively (*P* = 0.11). For depression symptoms, there were no changes from pre- to postoperative.

Concomitant nasal surgery was performed in 97 of 228 patients. A subgroup analysis showed that nasal surgery did not influence outcomes regarding AHI, ODI, ESS, or snoring (see Online Resource 4).

Preoperative AHI increased with each Friedman tongue grade by 5.4 ± 1.5/h (*P* = 0.008). However, no significant relationship between AHI reduction and Friedman tongue position was found. No association of pre- or postoperative AHI nor with its reduction through surgery was found in all other head and neck examination findings, including Friedman Staging System of OSA, septal deviation and turbinate hypertrophy, tongue base hypertrophy, epiglottis configuration, soft palatal webbing, the distance of the soft palate to the posterior pharyngeal wall, uvula configuration, dental status, tooth position (Angle 1–3) or bruxism.

### Adverse events and patient satisfaction

26% (60/228) of patients reported any adverse events after surgery. A foreign body sensation was the most frequent complaint reported by 38 patients, followed by difficulty swallowing in 20 patients. These symptoms were mainly temporary; only 26 patients reported symptoms after 3 months. 91% (109/120) of patients would undergo surgery again, and 94% (110/117) recommend it to a friend.

### Lost to follow-up

Patients who were lost to follow-up were younger (39.9 ± 11.8 years vs. 46.2 ± 11.0 years, *P* < 0.001) and had significantly lower preoperative AHI (lost to follow-up 17.3 ± 17.3/h vs. followed 26.1 ± 18.8/h, *P* < 0.001). No significant difference between the lost to follow-up and followed patients was found regarding tonsil grade (*P* = 0.40), tonsil volume (followed 5.9 ± 3.6 vs. lost 6.5 ± 3.7, *P* = 0.07), sex (*P* = 0.55) or BMI (followed 28.9 ± 4.4 kg/m^2^ vs. lost 27.8 ± 5.0 kg/m^2^, *P* = 0.12).

## Discussion

In this study, we expand our previous analysis of an earlier published cohort undergoing rfUPP ± TE in a larger patient group and more detailed patient characteristics [11]. The main differences to the previous study are that we included all severities of sleep-disordered breathing and patients with prior TE undergoing isolated rfUPP. The larger sample size eliminates previously underpowered analyses and allows for more robust conclusions.

We found a good correlation between intraoperatively measured tonsil volume and tonsil grade on the clinical examination, even after controlling for confounders. Tonsil volume was higher in men and increased BMI but decreased with age. The effects of age and BMI are minor but statistically significant. Compared to a previous analysis, the measured volume increases with each grade, indicating that tonsil volume can be well-predicted with clinical examination. The intramural part of the tonsils is not visible on clinical examination and might account for some of the observed variability. Mengi et al. could demonstrate a good correlation of tonsil volume with transcervical sonography measurements, indicating that sonography might aid in better estimating the tonsil volume than clinical examination alone [21].

Tonsil grade and volume are significantly associated with increased preoperative AHI and a greater reduction in AHI through rfUPP ± TE. Age, BMI, and neck circumference were positively correlated with pre- and postoperative AHI but did not influence the reduction through surgery. Even after controlling for these variables, tonsil volume and grade could predict preoperative AHI and AHI reduction. The results for ODI follow the same relationships with predictors as AHI. For daytime sleepiness and snoring, we found only a trend toward higher tonsil grades resulting in better outcomes of palatal surgery. Daytime sleepiness is more likely influenced by additional factors such as arousal threshold and disruption of sleep architecture and, therefore, subject to greater variability. Snoring perception is also subject to other factors such as loudness, regularity, throatiness, and assessment is mainly in the beholders’ ear. In our cohort, no other parameters in the history or physical examination predicted outcomes of AHI, daytime sleepiness, or snoring.

Outcome studies of UPPP ± TE rarely report tonsil grade or measure volume. Our results demonstrate that this is a crucial factor in predicting outcomes. The lack of reported tonsil size is a major limitation when comparing different techniques of palatal surgery or other interventions for sleep-disordered breathing. Without the knowledge of tonsil size, study results are not directly applicable to the individual patient. Several studies observed tonsil size to be correlated with preoperative OSA severity [8–10]. However, we could find only one study investigating the role of tonsil size on the outcome of UPPP ± TE, which describes a significant correlation between surgical outcomes and tonsil volume but not the tonsil grade [12]. A meta-analysis by Camacho et al. and a recent randomized clinical trial by Sundman et al. found that isolated TE without palatal surgery can effectively reduce AHI, indicating that the removal of tonsils might be a crucial part of a UPPP procedure [22, 23]. They also remark that few authors systematically remove smaller tonsils if they are still present, leading to biased outcome studies. A review by Maurer showed that success rates double if a tonsillectomy is performed in addition to a UPPP [24]. In the present study, all tonsils were removed irrespective of size and OSA severity, eliminating any bias toward positive results by removing only large tonsils. Several and sometimes conflicting predictors for success are reported in the literature, such as tongue position [13], Friedman staging of OSA [16, 25], age[13], and hyoid position.[25, 26] Besides the anatomical description of the upper airway, a neuromuscular component may influence outcomes but is hard to quantify and remains elusive [27]. Drug-induced sleep endoscopy offers a dynamic evaluation. A study by Chui et al. found a complete concentric collapse of the soft palate to be a negative predictor for outcomes of UPPP without TE [28]. Finding surgical success predictors is complicated by the large variability in preoperative factors and considerable night-to-night variability of AHI [29]. Interestingly, the AHI of some patients with absent or small tonsils deteriorated in our study. This might not be an actual deterioration but rather due to the night-to-night variability in patients with mostly low baseline AHI values [29]. This inherent variability in sleep medicine means that large cohort sizes are required for adequately powered statistical analyses, which are rarely found in surgical outcome studies. In our pilot study with 70 patients, the preoperative AHI increased by 1.4 s/h per ml of tonsil volume, whereas, in the present study, this was only 0.8/h per ml based on a much larger cohort. Moreover, tonsil volumes between grades I and II did not differ significantly in the pilot study, which is the case with the present cohort. In our opinion, the data set of this study allows for the most reliable assessment of the impact of tonsil size and volume on AHI, ODI, BMI, and age. Knowledge of the most decisive factors on the outcome of UPPP ± TE is crucial when counseling patients before this procedure. Thus far, tonsil size, if measured, is frequently among the strongest predictors of success [12, 13]. Interestingly, patients with absent tonsils undergoing an isolated rfUPP did not show a reduction in postoperative AHI, and some patients experienced a deterioration after surgery.

Our study has several limitations. The cohort consisted of predominantly male and middle-aged patients with BMI ranging from normal to moderately overweight. Confounders of sleep testing such as alcohol, sleep medication, nicotine, and caffeine intake were not directedly controlled. However, subjects were counseled to follow their habitual routines for every measurement to achieve comparable results. In all patients, radiofrequency ablation of the soft palate and incision of the palatopharyngeal arch was performed, limiting variability and allowing for a homogenous cohort. The results from the radiofrequency UPP might not be generalizable to other types of soft palate surgeries, including more conventional UPPP using suturing techniques. A recent randomized controlled trial showed radiofrequency UPP without TE and UPPP with TE were both effective in reducing daytime sleepiness and AHI. However, the AHI responder rate in moderate OSA was significantly lower for rfUPP without TE than conventional UPPP with TE (30% vs. 77%) [30]. We believe that tonsillectomy was the decisive factor for the difference in the outcome.

We included patients with nasal surgery in the analysis, since several studies have shown that nasal surgery does improve nasal obstruction but does not systematically affect OSA results [13, 31, 32]. Nasal surgery did not significantly affect surgical outcomes in our cohort. However, concomitant nasal surgery might introduce additional variability due to relatively unpredictable effects on sleep-disordered breathing.

A substantial proportion of patients was lost to follow-up. Patients lost to follow-up were younger and with a less severe OSA at baseline but were not different regarding tonsil size and volume from those included. Our results might be negatively biased, since patients with a resolution of their symptoms are hypothetically less likely to come for a follow-up visit. With a follow-up after 3 months, the current study cannot estimate the long-term results, which might deteriorate over time [7]. Overall, rfUPP ± TE improved respiratory parameters, snoring, and daytime sleepiness with high patient satisfaction and few adverse effects.

## Conclusion

Intraoperatively measured tonsil volume strongly correlates with tonsil size on clinical examination. rfUPP ± TE is an effective surgical treatment for reducing respiratory parameters, snoring, and daytime symptoms. Tonsil size on clinical examination or intraoperatively measured volume are good predictors for postoperative AHI and ODI reduction. In contrast, relief of snoring and daytime sleepiness improves irrespective of removed tonsil volume. When counseling patients before UPPP ± TE, surgeons should know that tonsil size and volume are the most crucial factor for AHI reduction. However, they should also keep in mind that improvement of daytime sleepiness and snoring control, which might be even more important than AHI reduction for some patients, does not depend on the volume of removed tonsil tissue.

## Supplementary Information

Below is the link to the electronic supplementary material.

Supplementary file1 (PDF 49 KB)

Supplementary file2 (PDF 57 KB)

Supplementary file3 (PDF 70 KB)

Supplementary file4 (PDF 48 KB)

## Data Availability

Data will be made available upon reasonable request to the corresponding author.

## References

[1] Heinzer R, Vat S, Marques-Vidal P, Marti-Soler H, Andries D, Tobback N, Mooser V, Preisig M, Malhotra A, Waeber G, Vollenweider P, Tafti M, Haba-Rubio J (2015). Prevalence of sleep-disordered breathing in the general population: the HypnoLaus study. Lancet Respir Med.

[2] Gottlieb DJ, Yenokyan G, Newman AB, O’Connor GT, Punjabi NM, Quan SF, Redline S, Resnick HE, Tong EK, Diener-West M, Shahar E (2010). Prospective study of obstructive sleep apnea and incident coronary heart disease and heart failure: the sleep heart health study. Circulation.

[3] Yaggi HK, Concato J, Kernan WN, Lichtman JH, Brass LM, Mohsenin V (2005). Obstructive sleep apnea as a risk factor for stroke and death. N Engl J Med.

[4] Patel SR, Bakker JP, Stitt CJ, Aloia MS, Nouraie SM (2021). Age and sex disparities in adherence to CPAP. Chest.

[5] Browaldh N, Nerfeldt P, Lysdahl M, Bring J, Friberg D (2013). SKUP ^3^ randomised controlled trial: polysomnographic results after uvulopalatopharyngoplasty in selected patients with obstructive sleep apnoea. Thorax.

[6] Fujita S, Conway W, Zorick F, Roth T (1981). Surgical correction of anatomic azbnormalities in obstructive sleep apnea syndrome: uvulopalatopharyngoplasty. Otolaryngol-Head Neck Surg Off J Am Acad Otolaryngol-Head Neck Surg.

[7] Stuck BA, Ravesloot MJL, Eschenhagen T, de Vet HCW, Sommer JU (2018). Uvulopalatopharyngoplasty with or without tonsillectomy in the treatment of adult obstructive sleep apnea—a systematic review. Sleep Med.

[8] Cahali MB, de Soares CFP, da Dantas DAS, Formigoni GGS (2011). Tonsil volume, tonsil grade and obstructive sleep apnea: is there any meaningful correlation?. Clinics.

[9] Jara SM, Weaver EM (2018). Association of palatine tonsil size and obstructive sleep apnea in adults: tonsil size and sleep apnea in adults. Laryngoscope.

[10] Lai C-C, Friedman M, Lin H-C, Wang P-C, Hsu C-M, Yalamanchali S, Lin M-C, Chen Y-C (2014). Objective versus subjective measurements of palatine tonsil size in adult patients with obstructive sleep apnea/hypopnea syndrome. Eur Arch Otorhinolaryngol.

[11] Tschopp S, Tschopp K (2019). Tonsil size and outcome of uvulopalatopharyngoplasty with tonsillectomy in obstructive sleep apnea: tonsil size and outcome of UPPP with TE. Laryngoscope.

[12] Matarredona-Quiles S, Carrasco-Llatas M, de Apodaca PM-R, Ortega-Beltrá N, Dalmau-Galofre J (2022). Is there a relationship between tonsil volume and the success of pharyngeal surgery among adult patients with obstructive sleep apnea?. Braz J Otorhinolaryngol.

[13] Choi JH, Lee JY, Cha J, Kim K, Hong S-N, Lee SH (2017). Predictive models of objective oropharyngeal OSA surgery outcomes: Success rate and AHI reduction ratio. PLoS ONE.

[14] von Elm E, Altman DG, Egger M, Pocock SJ, Gøtzsche PC, Vandenbroucke JP (2007). Strengthening the reporting of observational studies in epidemiology (STROBE) statement: guidelines for reporting observational studies. BMJ.

[15] Brodsky L, Moore L, Stanievich JF (1987). A comparison of tonsillar size and oropharyngeal dimensions in children with obstructive adenotonsillar hypertrophy. Int J Pediatr Otorhinolaryngol.

[16] Friedman M, Ibrahim H, Bass L (2002). Clinical staging for sleep-disordered breathing. Otolaryngol Neck Surg.

[17] Johns MW (1991). A new method for measuring daytime sleepiness: the Epworth sleepiness scale. Sleep.

[18] Powles WE (1974). Beck, Aaron T. *Depression: Causes and Treatment.* Philadelphia: University of Pennsylvania Press, 1972. Pp. 370. $4.45. Am J Clin Hypn.

[19] Morin CM, Belleville G, Bélanger L, Ivers H (2011). The insomnia severity index: psychometric indicators to detect insomnia cases and evaluate treatment response. Sleep.

[20] Sher AE, Schechtman KB, Piccirillo JF (1996). The efficacy of surgical modifications of the upper airway in adults with obstructive sleep apnea syndrome. Sleep.

[21] Mengi E, Sağtaş E, Kara CO (2020). Assessment of tonsil volume with transcervical ultrasonography in both children and adults. J Ultrasound Med.

[22] Camacho M, Li D, Kawai M, Zaghi S, Teixeira J, Senchak AJ, Brietzke SE, Frasier S, Certal V (2016). Tonsillectomy for adult obstructive sleep apnea: a systematic review and meta-analysis: adult tonsillectomy for OSA. Laryngoscope.

[23] Sundman J, Nerfeldt P, Fehrm J, Bring J, Browaldh N, Friberg D (2022). Effectiveness of tonsillectomy vs modified uvulopalatopharyngoplasty in patients with tonsillar hypertrophy and obstructive sleep apnea: the TEAMUP randomized clinical trial. JAMA Otolaryngol Neck Surg.

[24] Maurer JT (2009). Update on surgical treatments for sleep apnea. Swiss Med Wkly.

[25] Choi JH, Cho SH, Kim S-N, Suh JD, Cho JH (2016). Predicting outcomes after uvulopalatopharyngoplasty for adult obstructive sleep apnea: a meta-analysis. Otolaryngol Neck Surg.

[26] Millman RP, Carlisle CC, Rosenberg C, Kahn D, McRae R, Kramer NR (2000). Simple predictors of uvulopalatopharyngoplasty outcome in the treatment of obstructive sleep apnea. Chest.

[27] Zhao D, Li Y, Qu Y, Zhang J, Cao X, Ye J (2020). The role of genioglossus activity in predicting uvulopalatopharyngoplasty outcomes. Otolaryngol Neck Surg.

[28] Chiu F-H, Chen C-Y, Lee J-C, Hsu Y-S (2021). Effect of modified uvulopalatopharyngoplasty without tonsillectomy on obstructive sleep apnea: polysomnographic outcome and correlation with drug-induced sleep endoscopy. Nat Sci Sleep.

[29] Tschopp S, Wimmer W, Caversaccio M, Borner U, Tschopp K (2021). Night-to-night variability in obstructive sleep apnea using peripheral arterial tonometry: a case for multiple night testing. J Clin Sleep Med.

[30] Amali A, Motiee-Langroudi M, Saedi B, Rahavi-Ezabadi S, Karimian A, Amirzargar B (2017). A comparison of uvulopalatopharyngoplasty and modified radiofrequency tissue ablation in mild to moderate obstructive sleep apnea: a randomized clinical trial. J Clin Sleep Med.

[31] Johnson DM, Soose RJ, Lin H-C (2017). Updated nasal surgery for obstructive sleep apnea. Advances in oto-rhino-laryngology.

[32] Koutsourelakis I, Georgoulopoulos G, Perraki E, Vagiakis E, Roussos C, Zakynthinos SG (2008). Randomised trial of nasal surgery for fixed nasal obstruction in obstructive sleep apnoea. Eur Respir J.

