# Supplementary figures and images for: Tonsil volume and outcome of radiofrequency uvulopalatoplasty with or without tonsillectomy in adults with sleep-disordered breathing
Source: Eur Arch Otorhinolaryngol. 2023 Mar 12;280(6):3005–13. doi: 10.1007/s00405-023-07914-0 (PMC10175372; doi:10.1007/s00405-023-07914-0)

**Online Resource 1** Preoperative apnea-hypopnea index depending on tonsil volume (n=307)

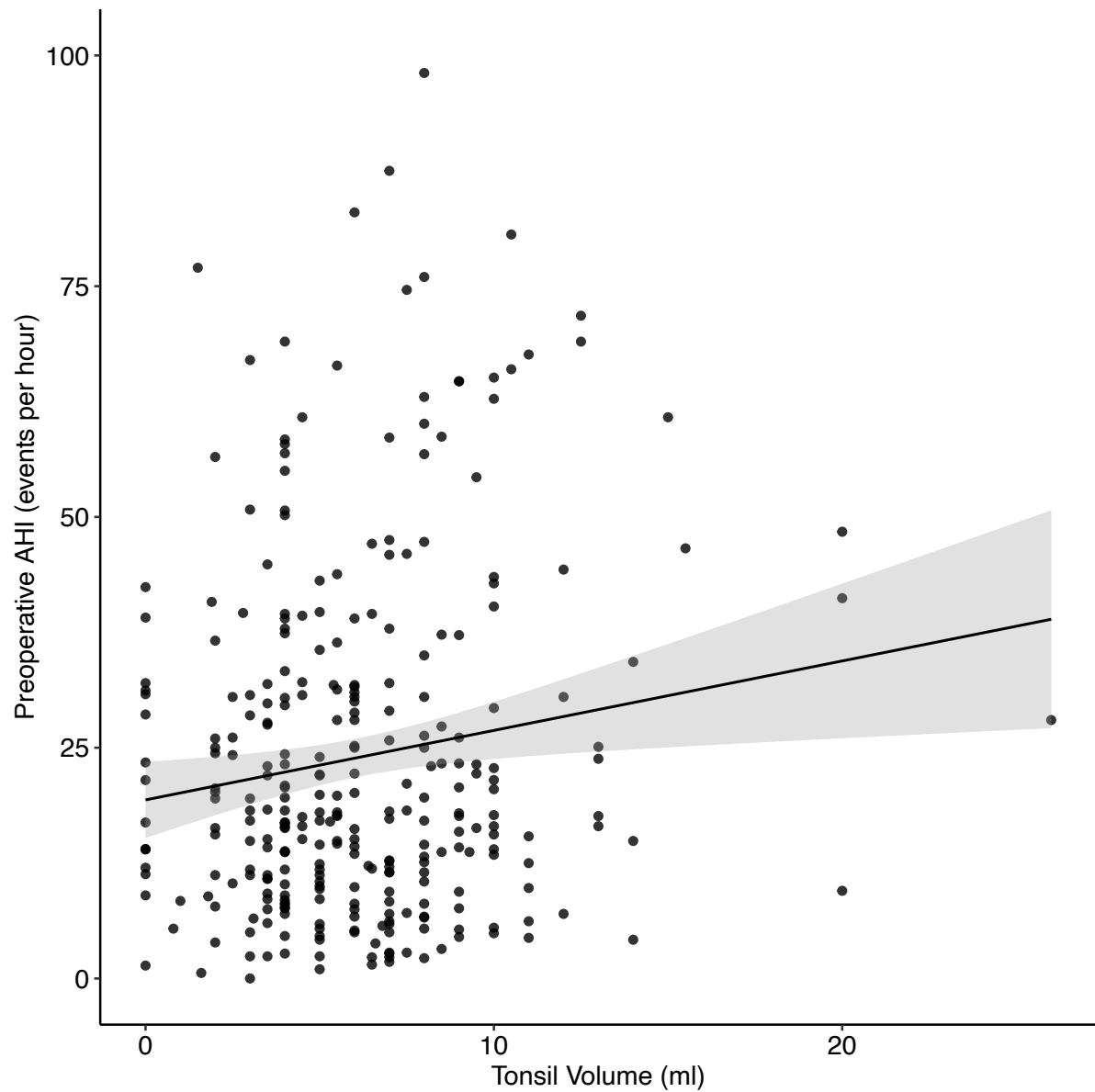

Supplement: Supplementary file 1 — Supplementary file1 (PDF 49 KB) [file 405_2023_7914_MOESM1_ESM.pdf]

**Online Resource 2** Preoperative apnea-hypopnea index depending on tonsil grade (n=307)

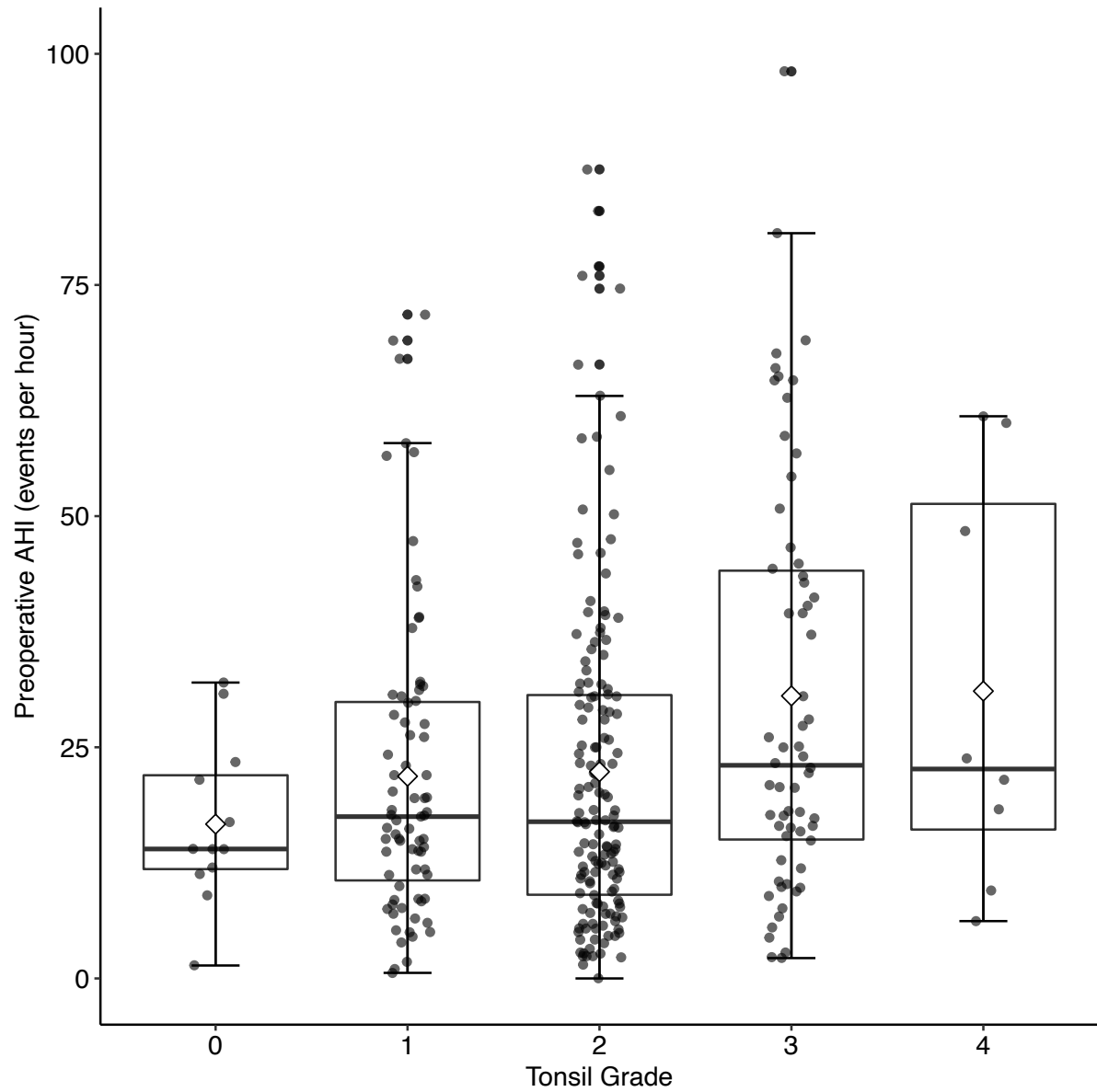

Supplement: Supplementary file 2 — Supplementary file2 (PDF 57 KB) [file 405_2023_7914_MOESM2_ESM.pdf]
