# Supplementary material for: Tonsil volume and outcome of radiofrequency uvulopalatoplasty with or without tonsillectomy in adults with sleep-disordered breathing
Source: Eur Arch Otorhinolaryngol. 2023 Mar 12;280(6):3005–13. doi: 10.1007/s00405-023-07914-0 (PMC10175372; doi:10.1007/s00405-023-07914-0)
Supplement: Supplementary file 3 — Supplementary file3 (PDF 70 KB) [file 405_2023_7914_MOESM3_ESM.pdf]

Online Resource 3 A comparison of responder and non-responder for apnea-hypopnea index (AHI) reduction according to Sher

	Non-Respnder	Responder	P Value
No. of patients	132	96	
Tonsil volume (mL)	5.2 (2.8)	7.0 (4.1)	<0.01
Gender, female	14 (10.6)	12 (12.5)	0.82
Age, years	47.0 (10.9)	44.9 (10.8)	0.15
Height (cm)	177.4 (8.6)	176.0 (8.1)	0.24
Weight (kg)	90.8 (14.9)	90.0 (15.1)	0.7
Body mass index (kg/m2)	28.8 (4.1)	29.1 (4.8)	0.7
Neck circumference (cm)	41.6 (4.0)	41.8 (3.1)	0.67
American Society of Anesthesiologists Classification (%)			0.21
1	12 (13.0)	9 (13.6)	
2	70 (76.1)	55 (83.3)	
3	10 (10.9)	2 (3.0)	
Friedman Stage of OSA (%)			0.79
1	3 (3.0)	1 (1.4)	
2	48 (48.5)	34 (47.9)	
3	47 (47.5)	34 (47.9)	
4	1 (1.0)	2 (2.8)	
**Apnea-hypopnea index (events/hour)**			
Preoperative	23.7 (19.9)	29.4 (17.0)	0.02
Postoperative	22.0 (16.7)	7.8 (5.1)	<0.01
Reduction absolute	1.7 (13.4)	21.5 (14.3)	<0.01
Reduction relative	9.4 [-46.8, 38.3]	71.0 [61.8, 81.7]	<0.01
Responder	0 (0.0)	96 (100.0)	<0.01
**Epworth Sleepiness Scale**			
Preoperative	8.9 (4.9)	9.3 (4.8)	0.51
Postoperative	4.2 (3.0)	3.5 (2.9)	0.15
Reduction absolute	4.3 (4.3)	6.0 (5.0)	0.05
Reduction relative	50.0 [19.4, 76.4]	62.5 [33.3, 84.2]	0.1
Responder	33 (55.0)	41 (70.7)	0.12
**Snoring index (VAS 0-10)**			
Preoperative	8.0 [7.0, 9.0]	8.5 [7.0, 9.8]	0.39
Postoperative	3.0 [2.0, 5.0]	3.0 [2.0, 4.0]	0.96
Reduction absolute	6.0 [3.5, 7.0]	5.0 [4.0, 6.2]	0.91
Reduction relative	66.7 [47.2, 80.0]	61.2 [50.0, 72.3]	0.37
Responder	30 (57.7)	26 (55.3)	0.97
