# Supplementary material for: Tonsil volume and outcome of radiofrequency uvulopalatoplasty with or without tonsillectomy in adults with sleep-disordered breathing
Source: Eur Arch Otorhinolaryngol. 2023 Mar 12;280(6):3005–13. doi: 10.1007/s00405-023-07914-0 (PMC10175372; doi:10.1007/s00405-023-07914-0)
Supplement: Supplementary file 4 — Supplementary file4 (PDF 48 KB) [file 405_2023_7914_MOESM4_ESM.pdf]

Online Resource 4 A comparison of patients with and without concomitant nasal surgery

	No Nasal Surgery	Nasal Surgery	P Value
No. of patients	131	97	
Apnea-hypopnea index (events/hour)			
Preoperative	24.9 (19.7)	22.6 (17.5)	0.29
Postoperative	16.1 (15.3)	16.0 (14.4)	0.95
Reduction absolute	10.5 (17.5)	9.4 (16.1)	0.62
Responder	56 (42.4)	40 (41.2)	0.96
Epworth Sleepiness Scale			
Preoperative	8.1 (4.4)	9.1 (5.1)	0.1
Postoperative	4.2 (3.1)	3.6 (2.6)	0.26
Reduction absolute	4.5 (4.4)	5.3 (5.0)	0.33
Responder	38 (52.1)	40 (69.0)	0.08
Snoring index (VAS 0-10)			
Preoperative	8.0 [7.0, 9.5]	8.0 [7.0, 9.0]	0.52
Postoperative	3.0 [2.0, 5.0]	3.0 [2.0, 4.0]	0.25
Reduction absolute	5.0 [4.0, 6.0]	5.0 [4.0, 6.5]	0.9
Responder	31 (51.7)	30 (57.7)	0.57
